# A Brain-Controlled Quadruped Robot: A Proof-of-Concept Demonstration

**DOI:** 10.3390/s24010080

**Published:** 2023-12-22

**Authors:** Nataliya Kosmyna, Eugene Hauptmann, Yasmeen Hmaidan

**Affiliations:** 1 Media Lab, Massachusetts Institute of Technology, Cambridge, MA 02139, USA; 2 Reactive Lions Inc., San Francisco, CA 94105, USA; 3 Psychology Department, University of Toronto, Toronto, ON M5S 3E4, Canada; yasmeen.hmaidan@mail.utoronto.ca

**Keywords:** brain–computer interfaces, robots, personal assistance, artificial intelligence, electroencephalography (EEG), electrooculography (EOG), wearables

## Abstract

Coupling brain–computer interfaces (BCIs) and robotic systems in the future can enable seamless personal assistant systems in everyday life, with the requests that can be performed in a discrete manner, using one’s brain activity only. These types of systems might be of a particular interest for people with locked-in syndrome (LIS) or amyotrophic lateral sclerosis (ALS) because they can benefit from communicating with robotic assistants using brain sensing interfaces. In this proof-of-concept work, we explored how a wireless and wearable BCI device can control a quadruped robot—Boston Dynamics’ Spot. The device measures the user’s electroencephalography (EEG) and electrooculography (EOG) activity of the user from the electrodes embedded in the glasses’ frame. The user responds to a series of questions with YES/NO answers by performing a brain-teaser activity of mental calculus. Each question–answer pair has a pre-configured set of actions for Spot. For instance, Spot was prompted to walk across a room, pick up an object, and retrieve it for the user (i.e., bring a bottle of water) when a sequence resolved to a YES response. Our system achieved at a success rate of 83.4%. To the best of our knowledge, this is the first integration of wireless, non-visual-based BCI systems with Spot in the context of personal assistant use cases. While this BCI quadruped robot system is an early prototype, future iterations may embody friendly and intuitive cues similar to regular service dogs. As such, this project aims to pave a path towards future developments in modern day personal assistant robots powered by wireless and wearable BCI systems in everyday living conditions.

## 1. Introduction

The robotics industry is on the rise, with robots taking an active part in many environments: museums [1], fashion shows [2], hotels [3], classrooms [4], warehouses [5], and military use cases [6]. While some of the use cases such as military and warehouses are being actively explored and reported alongside multimillion dollar investments, personal assistant mobile robots that are catering specifically for the needs of the users but are also able to support the user by bringing the groceries in, watering a plant, or opening a door are practically non-existent. Additionally, a lot of control modalities for those robots include almost exclusively voice or touch interfaces, and users who cannot manipulate those robots using those modalities are being given an option to control the desired system using their brain activity. These systems are more commonly known as brain–computer interfaces (BCIs). In a lot of cases, users who have looked-in syndrome (LIS) or amyotrophic lateral sclerosis (ALS), among other conditions, benefit the most from an ability to control a robot using a BCI. However, most systems designed for these users include wheelchair or exoskeleton controls [7,8]. Moreover, to control the wheelchair in most cases, the user would still need to rely on the gaze or presence of visual stimuli—either directly measured using eye tracking or electrooculography (EOG), or indirectly by using event-related potentials (ERPs), an activation in a certain area of the brain in response to a stimulus (event) [9]. Additionally, in most cases, these BCI systems are coupled with gel-based caps that host the electrodes, or are implantable solutions, thus making the interaction rarely attempted in the real world, or easily scalable due to costs involved in surgery [8].

In this paper, we want to bridge the gap by demonstrating a proof-of-concept (PoC) system of an assistive robot, controlled using a wearable, wireless BCI. Our PoC uses (1) non-visual-based mental tasks for control and (2) a mobile robot that can support the user by carrying the items as well as being able to bring a chair or open a door. Unlike exoskeletons, such a solution could be deployed for users who prefer/require being immobile. Additionally, we want to ensure that both (1) and (2) parts of the system are mobile, wireless, and autonomous. We thus chose to use a pair of wireless glasses with EEG/EOG electrodes [10,11], embedded in the frame of the glasses. We found this form-factor to be promising and novel, as well as it to be the least intrusive means possible for the user. We also selected Spot, a mobile robot from Boston Dynamics [12], as the prototype. Using an iPhone, the user responds to a series of questions with YES/NO answers, by performing a brain-teaser activity of mental calculus, while wearing the glasses. Each question–answer pair has a pre-configured set of actions for Spot. For example, Spot was prompted to walk across a room to another area of the room, pick up an object, and retrieve it for the user (i.e., bring a bottle of water) when a sequence resolved to a YES answer. A total of 83.4% accuracy was achieved. Though Spot is currently priced at USD 130K as of writing this paper, it is the only agile robot capable of effectively executing heavy duty tasks on behalf of the user as well as carrying out other tasks in human-centric environments. It is also a unique solution in terms of our needs of mobility. To the best of our knowledge, this is the first integration of wireless, non-visual-based BCI systems with Spot in the context of personal assistant use cases.

The rest of our paper is organized as follows: we briefly describe the current state of the art in BCIs and robotics, as well as the most common use cases, moving to an in-depth overview of the infrastructure of the system, introducing the task and preliminary results of the study, before highlighting challenges, limitations, and future work.

## 2. State of the Art

### 2.1. BCI Paradigms Used in Robotics Applications

Over the past 20 years there has been an active exploration of the possibility of controlling different robots and neuroprosthetic devices using non-invasive electroencephalography (EEG), which captures the brain signals from electrodes placed on a scalp [13,14,15,16,17]. EEG to this day remains one of the most practical and applicable non-invasive BCI methods because other imaging modalities such as functional magnetic resonance imaging (fMRI), magnetoencephalography (MEG), and positron emission tomography (PET) are expensive [18], technically demanding, and not easily portable, though a lot of progress on reducing their cost and introducing portable solutions has been reported on recently.

A BCI system can be controlled using either endogenous (spontaneous) or exogenous (evoked) signals. In exogenous BCI, evoked signals appear when a person pays attention to an external stimulus such as a visual or auditory cue. The advantage of this approach includes minimalistic training as well as high bit rates up to 60 bits/min [19,20]. However, the user needs to always attend to the stimuli, which limits its applicability in real-life conditions. Also, the user can become quickly tired when using exogenous BCIs. Typical examples include modulations of steady-state-evoked potentials (SSEP) and P300 [18]. In endogenous BCIs, on the other hand, control signals are generated independently from any external stimulation and can be fully executed by the user on demand. It is also useful for those users who have sensory impairments while providing a more natural and intuitive means of interactions since users can spontaneously issue commands to the system [18]. It typically requires longer training sessions, and the bit rate is reportedly lower. Examples include motor imagery (MI) [21]. Motor imagery, however, is considered a complex, cognitively demanding task [22], and thus other mental strategies for BCI control are being actively explored, such as imagining music [23], phoneme imagery [24], visual imagery [25], mental rotation, mental calculations, and word association [26].

**Figure 1:**
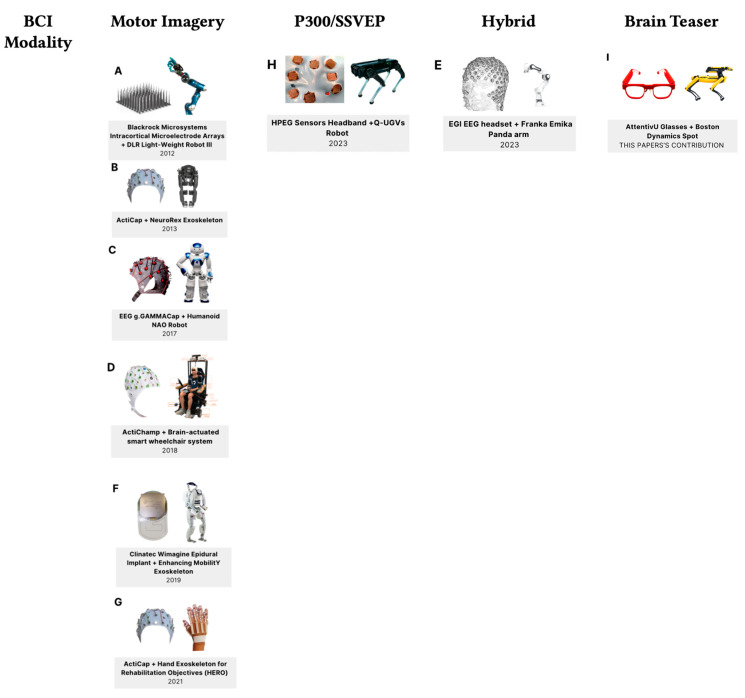
The overview of the state of the art in BCI and robotics as of 2023. Selected papers are featured, representative of the directions of the research, including (**A**) [29] and (**F**) [8]—the implants used to control limbs and exoskeleton using MI BCI paradigm; (**E**) [30]—the most recent paper to date, featuring a hybrid MI + SSVEP + EMG system; (**B**) [16], (**D**) [31], and (**G**) [32], all using the same cap for different robotic use cases as well as the MI BCI paradigm; (**C**) [33] featuring the P300 interface; and finally, (**H**) [34], the only paper to the best of our knowledge featuring a quadruped robot and using the SSVEP BCI paradigm. All users trained on these aforementioned systems also required a visual-based training protocol. The figure also illustrates the challenges of portability and comfort for the users who would wear BCIs, with our proposed solution in this paper (**I**) being the only truly wearable form-factor with a setup time under 2 min.

In this paper, we explore mental calculation—a mental task that requires problem-specific mental work, but no presence of stimuli is needed, unlike exogenous tasks, and therefore it is sometimes referred to as a ‘brain teaser’ [26]. For this task, the ease-scale showed an increasing trend over sessions, which indicates that practice might also reduce the workload in this task, unlike strategies like MI [26]. To the best of our knowledge, we have not been able to identify a brain-actuated robotic system that involved both visual-free stimuli training and control tasks using ‘brain teaser’ strategies (see Figure 1). Most papers make use of the motor imagery (MI) paradigm, which is considered cognitively demanding, while several others feature SSVEP or P300 paradigms, which are visual paradigms. We thus believe that exploring other, non-visual paradigms might be beneficial for the brain-actuated robot control, due to the increased wearability of the device itself (there is no need to position electrodes on the pre-motor and motor cortex, which requires a cap design, but more of a headband design is sufficient); ‘brain teaser’ tasks usually do not require any visual training, and they are cognitively less demanding for the participants [26].

### 2.2. Robotics Applications Enabled by BCIs

The most commonly used robotics applications that make use of BCIs are usually for populations that require assistance, and they often include wheelchairs and exoskeletons. Figure 1 shows a general overview of the state of the art of robotics and BCIs. We further refer the reader to the review paper by Zhang and Wang [27], a comprehensive review of robots controlled by motor imagery BCIs, including mobile robots and robotic arms.

#### 2.2.1. Wheelchairs

Smart wheelchairs generally have different functionalities that range from assistance to rehabilitation. Brain-actuated robot systems can extend these use cases further by making them accessible to those with LIS, ALS, and others. Galán et al. [28] proposed a 64 channel EEG-based BCI combined with mental tasks like left hand imagination movement, rest, and word association to drive a real and a simulated wheelchair along a predefined route. Some patients can also use hybrid BCIs to steer a wheelchair directly and not rely on autonomous navigation. In a study by Wang et al. [9], a combination of motor imagery, eye blinking, and P300 was used in a hybrid BCI system to move the wheelchair forward or backwards with four healthy subjects. Tonin et al. [21] also showed how participants with spinal cord injuries can be trained to control wheelchairs using the motor imagery paradigm. While these systems have a potential in helping people with ALS or LIS, they are currently limited to lab or hospital settings.

In order to adapt to a real-world setup, several labs are now turning towards BCI systems with a minimal number of electrodes and the signal processing hosted on mobile applications to provide the same wheelchair control functionality but within a mobile setup. In a study by Banach et al. [7], a healthy user wore a four EEG electrode system to steer a wheelchair. The user attempted to achieve a concentration state in a consecutive sequence determined by a codebook that tells the wheelchair to move front, back, left, or right depending on the number of concentration states achieved.

Tang et al. designed a P300-based brain-actuated omnidirectional wheelchair with an attached robotic arm, aiming for rehabilitation from spinal injury. Based on a real-time recognition module, a patient can trigger planned solutions for objects in a scene. For instance, the wheelchair can approach a door and deploy a robotic arm to open the door when a patient intends to go to the next room [31]. The system used image tracking, Kinect camera, and a P300 to detect which actions the patients want to target in their domestic environment. This system has a potential for everyday use because it is not limited to pre-programmed actions and uses object detection to respond to stimuli in the patient’s environment. Other systems like that of Na et al. [35] proposed an embedded lightweight SSVEP-BCI electric wheelchair with a hybrid stimulator.

#### 2.2.2. Exoskeletons

With a potential to offer more dexterous functionalities than wheelchairs, various types of exoskeletons have been actively developed and studied in recent years [28]. Exoskeletons are wearable robots exhibiting a close physical interaction with the human user. Among them, powered upper-limb and lower-limb exoskeletons have been given attention as the potential technology to assist paraplegic and tetraplegic populations. Benabid et al. [8] reported a proof-of-concept system with a semi-invasive epidural electrocorticography (ECoG) implant and neuroprosthetic exoskeleton designed to enhance full-body mobility with sensory-motor cortex input. For instance, two ECoG recorders, 64 electrodes each, were implanted over the upper limb sensorimotor areas of the brain of one subject. The subject was first able to control a virtual avatar through a game, and then he was able to control a four-limb exoskeleton when performing mental tasks. One of the current major drawbacks of such a system is the cost of the exoskeleton as well as the extensive surgery rehabilitation time required for the patients who undergo it. Additionally, similar to wheelchair control, a lot of research labs are focusing on exploring non-invasive solutions that are cheaper and more scalable. Lee et al. [36] proposed a BCI system for real-time control of a powered lower-limb exoskeleton based on EEG signals recorded over the user’s sensorimotor cortical areas. The authors trained a binary decoder that can distinguish two different mental states based on MI, which is applied in a cascaded manner to control the exoskeleton in three different directions: walk front, turn left, and turn right. Five subjects were able to complete the three-way navigation task using brain signals while mounted in the exoskeleton.

#### 2.2.3. Humanoid Robots

Brain-controlled humanoid robots were explored as a potential solution for patients with LIS. Spataro et al. [33] reported on inviting four ALS patients and four healthy controls to use a NAO humanoid robot with the aim of reaching and grasping a glass of water. Using a P300 oddball paradigm, the command selected by the user was executed by a robot. Despite NAO being a humanoid robot, it has a small size that limits which orientations and positions it can access. Additionally, it cannot carry any significant weight, open a door, etc. Chae et al. [37] also used NAO to create a humanoid navigation system controlled with an asynchronous BCI. Users were able to navigate the robot through an indoor maze to a target. They were presented with photos captured by the robot’s perspective while traversing through stages of the maze. The users controlled its movements with left-hand, right-hand, or forward motor-imagery-based commands while wearing an EEG cap. Daeglau et al. [38] highlighted how humanoid robots can be useful for optimizing neurofeedback when individual differences, motivation, and social contexts are considered within motor imagery tasks designed to control a robot’s movements. Choi and Jo [39] have presented hybrid BCI protocols that leverage P300, SSVEP, and event-related desynchronization (ERD) for robotic control coordination. Sorbello et al. [40] showed preliminary humanoid robot navigation results for four ALS patients with locked-in syndrome and four healthy controls. The NAO humanoid robot could walk in six directions and grasp/give objects. All users could navigate the robot, whereas those with ALS performed better in the visual attention and intention tasks compared to the healthy controls.

#### 2.2.4. Arm Robots

Collinger et al., in 2012 [41], and Wodlinger et al., in 2015 [42], reported performing two studies with the same female subject with tetraplegia who had two 96 channel intracortical electrode arrays implanted in her left motor cortex. The first study, performed in 2012 [30], reported on the continuous translation, orientation, and one-dimensional grasping control of a prosthetic limb (seven degrees of freedom). The second study, from 2015 [42], expanded the scope of the control signal by also extracting hand-shape commands. Robust neural tuning to hand shaping was found, leading to ten-dimensional (10D) performance well above chance levels in all tests.

Hockberg et al. [29] also demonstrated how people with tetraplegia could control a prosthetic arm with an implanted 96 channel intracortical microelectrode array years after their central nervous system injury or brainstem stroke. Patients managed to guide the arm to drink coffee from a straw in a designated robotics workspace. To make BCI-controlled hand exoskeleton motor training solutions more accessible, Araujo et al. [32] created the Hand Exoskeleton for Rehabilitation Objectives (HERO) with a lightweight and low-cost 3D-printed hand model as a proof of concept. One healthy user was prompted to imagine hand movements with MI tasks aimed at extending and flexing their five fingers to control the ergonomic prosthetic hand with EEG non-invasively recorded from their primary motor cortex (M1).

Zhang et al. [30] proposed Neural Signal Operated Intelligent Robots (NOIR), a general-purpose brain–robot interface that allows humans to use their brain signals to control robots to perform daily activities, such as making a sandwich, ironing clothes, and petting a robot dog. Three healthy subjects tested the system using a 128 channel EGI system. A modular pipeline for decoding human-intended goals from EEG signals was implemented using SSVEP + MI + EMG: (a) What object to manipulate, decoded from SSVEP signals; (b) How to interact with the object, decoded from MI signals; (c) Where to interact, decoded from MI signals. A safety mechanism that captures muscle tension from jaw clench is used to confirm or reject decoding results. Two robots were used in the experiment: a Franka Emika Panda arm and a PAL Tiago robot.

#### 2.2.5. Quadruped Robots

Jia et al. [43] proposed a protocol for brain-controlled quadruped movement that could theoretically use non-invasive EEG with motor imagery. A user would imagine five types of movements to create an instinct-like axis of locomotion for the robot’s associated limbs. This BCI protocol was designed to help potential developers map EEG signals with real-time robotic flexion/extension movements bound by instruction sets since the field is still in its early stages.

Overall, quadruped robots are often used to support users in complex work environments or defense applications. One of the most famous quadruped robots is the Boston Dynamics Spot robot that can carry payloads up to 15 kg and iteratively map maintenance sites like tunnels [12]. The real estate and mining industries are also adopting quadruped robots, like Spot, to help with monitoring on job sites with complex logistics [12].

Spot was the robot of choice in our paper due to its ability to execute a range of tasks with heavy payloads, open/close doors, etc. We believe that Spot is the closest candidate to be used as an assistive robot for patients with ALS or LIS, and moreover, such a scenario has not been explored to date.

To the best of our knowledge, there is to date only one report of controlling quadruped robots using BCI. Faisal et al. [34] report on using an eight-channel EEG from the occipital region of the subject to control a Spot robot. The authors used HoloLens 2 as a device to both hold the sensors but also as a screen support to show flickering visual labels representing eight possible command choices so the user could make their selection using the SSVEP paradigm. The authors reported on the experiment with one user undergoing 24 trials. Though interesting, this paper is based on HoloLens 2 and SSVEP, and in our work, we explored the opportunities of using endogenous BCIs for robot control, mental calculus, in order to offer potential control to the users who cannot use their eye movements.

We now introduce the architecture of our system, which we refer to as Ddog: the Spot robot controlled using a mobile BCI solution, based on a mental calculus task.

## 3. Infrastructure of Ddog

The goal of the system was to design an autonomous application that would enable a user to control a Spot robot based on the user’s input via BCI and give feedback to the user and their caregiver using voice via the app.

**Figure 2:**
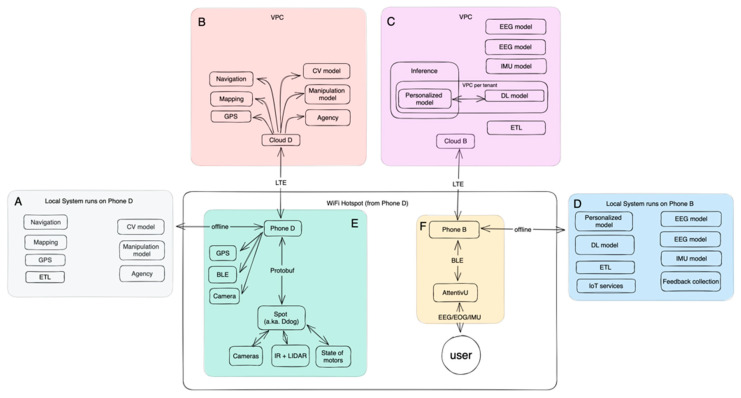
The architecture of the Ddog system.

**Figure 3:**
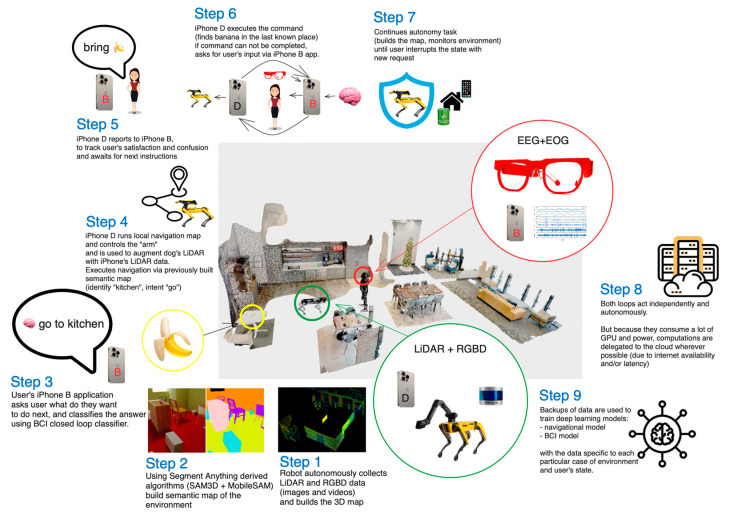
The overview of the entire system.

The system is designed to work either fully offline, or fully online. The online version has a more advanced set of machine learning (ML) models, as well as better fine-tuned models, while preserving the battery of the local devices. The system is designed to run in production, while allowing rapid iterations on the majority of the parts. The overview of the system can be seen on Figure 2 and Figure 3.

On the client side, the user interacts with the brain–computer interface (BCI) device (in the case of this paper, the AttentivU device (Cambridge, MA, USA) [10,11], to be introduced in depth in Section 4 of this paper), via a mobile application that communicates with the device using the Bluetooth low energy (BLE) protocol. The user’s mobile device (Phone B) communicates with another mobile phone that controls the Spot robot in order to enable agency, manipulation, navigation, and ultimately assistance for the end user. Communications between the mobile phones can happen via Wi-Fi or via the long-term evolution (LTE) protocol. There is a Wi-Fi hotspot that is established on the Phone D, and Ddog connects to it via Wi-Fi. The same happens with Phone B—it is connected to Phone D via the same Wi-Fi. At the same time, there is a possibility to run the system online, connected to the models running in the cloud independently.

There is a separation of concerns between the artificial intelligence (AI) models dedicated to sensitive biodata and AI models used for robotics and agency systems.

### 3.1. Server Side

The system was designed in a cloud-agnostic way. It runs in the Kubernetes (K8S) cluster, inside specially designed containers, called pods. Each cluster is deployed in its own virtual private cloud (VPC), and Cloud B has a separate VPC for each tenant.

### 3.2. Cloud D

This cloud works inside a dedicated VPC, and usually it is deployed in the same availability zone, closer to the end user, so the response latency is minimized for each service.

This cluster is represented by a group of containers (pods), each designed to serve a single purpose. In a way, it is an architecture of micro services, where each service is a running AI model, wherein the majority of them are ML models, with a few DL models, used to fine-tune models on the location and situation-specific data for each end-user.

These pods are responsible for (1) navigation; (2) mapping; (3) computer vision; (4) manipulation; (5) positioning; and (6) agency.

At the same time, the majority of the services are designed to run on the ‘edge’, which in this context means devices that are located on the premises (mobile phones, Spot robot, BCI device—AttentivU).

Let us go briefly over each service and what it does.

Mapping—a service to collect information about a robot’s surroundings from different sources. It maps static immovable data (a tree, a building, a wall), but also collects dynamic data that changes over time (a car, a person).

Navigation—based on the mapping data collected and augmented in the previous service, the navigation service is responsible for building the route between points A and B in space and time. It is also responsible for building alternative routes, if the previous route failed or changed, and it also assesses success associated with each route, as well as the estimated time needed for a robot to traverse it with the given payload (any extra objects the robot might carry).

Computer vision—this represents visual data collected from the cameras of the robot and augmented by data from the mobile phones, being used to generate the space and time representation. This service also tries to segment each visual point and recognize objects.

Manipulation—this service relies on mapping, navigation, and computer vision to enable the user’s intent of grabbing and transporting certain objects (like a bottle) or taking a particular position (not just a location, but also a position of the manipulator—arm, described below).

Positioning—this service uniquely maps the global positioning system (GPS) and in-door positioning data, together with the supplemented data from robot sensors: depth point clouds, light detection and ranging (LIDAR), infrared (IR) emission, etc. The positioning service compliments the mapping service, and it enables a set of common knowledge used to order navigation with items like room names, street names, and landmarks.

Agency—this service acts as a very simplified version of the intelligence built on the agency principle, and it ultimately enables Ddog to be an active assistant. Each agent collects and analyzes data based on the user’s intent, as well as data available from the public knowledge, but it is also based on the private data aggregated by the entire system. Agents are responsible for interacting with the user and invoking different scenarios such as ‘go to the kitchen’, ‘pick up a bottle’, and ‘bring a newspaper’.

### 3.3. Cloud B

Cloud B is responsible for training the models related to BCI, specifically, several different models: electroencephalography (EEG), electrooculography (EOG), and inertial measurement unit (IMU).

As in the previous example with Cloud D, the premise of Cloud B is to replicate an offline version of the system, which runs on the Phone B, and supplement it with a faster training pipeline and energy preservation.

Here is a brief overview of each particular model pipeline:

EEG model—this model aggregates features based on a ML model. The model used in this paper is described in Section IV of this paper.

EOG model—this model is used as an additional facet of biodata for each user, and it is used together with the EEG model.

IMU model—this model aggregates data about head and device movements and is the same as the EOG model; it is used to augment the EEG model.

Deep learning model—this model aggregates all the above three pre-trained models: EEG, EOG, IMU, to personalize accuracy and precision for each user.

ETL pipeline—this pipeline is responsible for aggregating and distributing data to each respective model continuously and according to existing regional regulations in regard to storing and processing biological data.

Inference—once each model is trained, it is deployed to run inferences and process real-time requests from the user. Each inference is deployed under a standalone VPC per tenant. Inference is optimized for latency and accuracy.

### 3.4. Client Side

**Figure 4:**
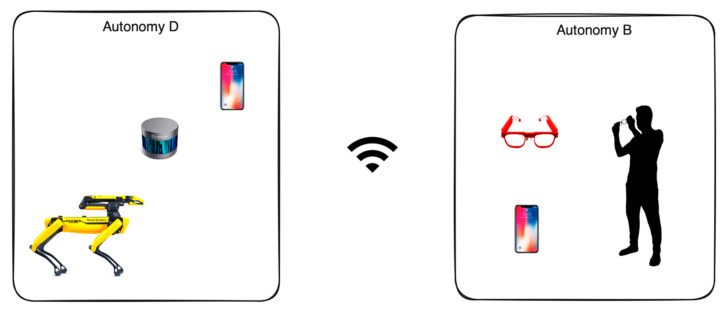
Visual representation of Cloud B and Cloud D.

The client side effectively runs on two mobile phones (Phone B and Phone D; Figure 4). Each phone replicates slower and energy-cautious functionality similar to how Cloud B and Cloud D function, respectively.

Phone D also collects relevant data like GPS and acts as an intermediary between the Ddog and Phone B by providing the network fabric via Wi-Fi hotspot functionality.

Phone B is responsible for communicating with the AttentivU device using BLE and addressing the user’s intent based on the state of the running agent.

Agency functionality is very limited in the offline mode because of the hardware limitations, and it performs best when deployed in Cloud D.

Here are two agents we implemented:

Navigational agent—an agent that executes navigational intents, like going to the kitchen or living room.

Manipulation agent—an agent responsible for recognizing an object, picking up the object, and bringing this object back to the user.

### 3.5. Computer Vision

The offline models deployed on the phone itself run data collection and aggregation, but also real-time inference using TensorFlow mobile models, that are optimized for a smaller RAM footprint and ARM-based CPUs.

We ran a bigger TensorFlow model in Cloud D that was optimized for higher accuracy at the expense of the bigger RAM footprint and much bigger energy consumption.

### 3.6. Data Collection

The offline system deployed on mobile phones is capable of navigating predefined cases autonomously using ML models deployed on Phone D.

We explored autonomy functionality provided by the Boston Dynamics (Waltham, MA, USA) team, specifically Map Data and Graph Nav APIs that are designed to function on top of Spot’s stack and build decision trees based on existing algorithms. While it is optimized to preserve the equipment and execute the goals to the best to its abilities, the API and functionality provided out of the box is lagging behind the functionality available on mobile phones and clouds. So, at the expense of reliability, we decided to go with the more advanced technological stack, compared to the one provided by Boston Dynamics, for the industrial use cases by default.

### 3.7. Segmentation

The original version we used to deploy segmentation models relayed on a single TensorFlow 3D model that leveraged LIDAR data. Later, we expanded models to few-shots models, augmented by supplementary models running neural radiance fields (NeRF) and RGBD data. We also employed TensorFlow and PyTorch, as well as more performant custom optimization written in C++ and Rust to be deployed on Phone D and Cloud D.

### 3.8. Depth, IR, LiDAR

The original data gathered by Ddog are aggregated from five cameras. Each camera can provide grayscale, fisheye, depth, and infrared data. There is also a sixth camera inside the arm’s gripper that has 4K resolution and LED functionality to provide enough resolution to detect objects using pre-trained existing models for TensorFlow.

This is another reason why we supplemented these data with the image data from Phone D that can also provide resolution up to 4K at 60 FPS or HD at 280 FPS.

Also, because we used Apple’s hardware for the phone, we leveraged LiDAR data collection to obtain depth and cloud points natively. The minimal hardware requirements are the iPhone 12 Pro, iPad Pro 11 (third generation), or iPad Pro 12.9 (fifth generation) (Apple, Cupertino, CA, USA).

### 3.9. Mapping

There is a basic mapping functionality that happens offline on Phone D; however, the Cloud D version aggregates all of the available data and builds maps for predefined locations.

#### 3.9.1. Points Cloud

Points clouds are generated from LiDAR data and RGBD data generated by Ddog and Phone D. After data are collected, they are normalized by a single coordinate system and matched to a global state that aggregates all the imaging and 3D positioning data.

#### 3.9.2. Imaging

As already stated above, the best images are collected from Phone D and Ddog’s arm, since they are the areas that can provide real-time data in 1080p resolution at 30 FPS, so our models were optimized for this specific quality.

When/if Ddog fails to execute navigation and manipulation commands, we collect additional data in 4K quality to improve particular location models.

#### 3.9.3. GPS

Because of Phone D’s hardware, we are able to collect GPS coordinates that are a must-have for maintenance and global positioning.

It is less useful in the apartment use case, but with enough resolution (1 m precision), we were able to leverage GPS data for the mapping.

### 3.10. Manipulation

**Figure 5:**
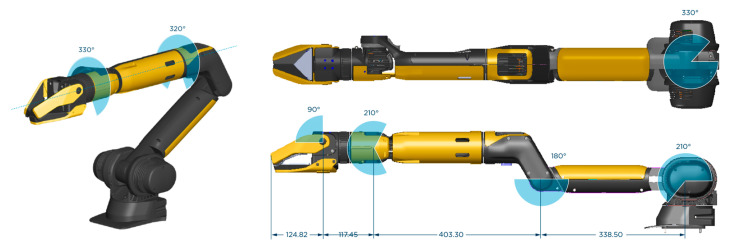
Arm and gripper for the Spot robot by Boston Dynamics.

Manipulation solely relies on the quality of the arm’s gripper installed on the Ddog (Figure 5); the one we used is manufactured by Boston Dynamics and optimized for industrial use cases of working with pipes and vents, though we found ways to leverage it for civilian, personal assistant use cases.

#### 3.10.1. Grips

After trying the gripper and manipulation functionality in different environments with a variety of over 40 different objects, we realized that the only way to build a reliable gripper would be to pre-train the model to work with well-known objects that we anticipate users to interact with.

**Figure 6:**
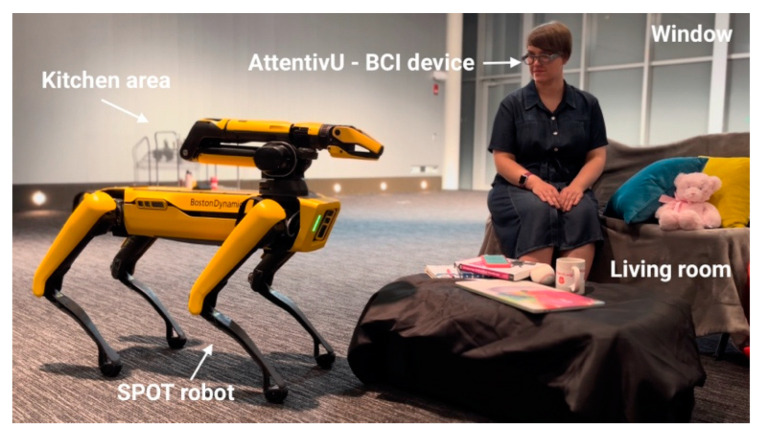
A person wearing AttentivU glasses about to perform a mental task of calculations in order to send Spot from the ‘living room’ space to the ‘kitchen space’.

To meet the goal of this paper, we limited use cases to basic interactions with the objects in the predefined locations: we mapped a big lab space to set it up as an ‘apartment’ with a ‘kitchen’ area (containing a tray of different cups and bottles), ‘living room’ area (small couch with pillows and a small coffee table), and ‘window lounge’ area. You can see all three areas in Figure 6. We enabled manipulation sequences with the chair, as well as plush toys. Finally, we enabled Spot to take a picture.

The biggest takeaway of working with the gripper is a sequence recording for different detected objects using different settings for sensitivity and force, executed by the arm. We urge readers to be careful when/if you try to replicate this kind of functionality, as it is inevitable that one are going to damage different objects (plastic bottles, cups, toys); thus, one must make sure they build the feedback loop, and a rapid ‘emergency stop’ button is always part of the UI/UX. It already works out of the box with Boston Dynamic’s API, as it is a fundamental robotics safety measure, and we encourage others to not ignore it by any means.

Because of the agency, we benefit from understanding the user’s intent better; by gaining a deeper understanding of what user anticipates to do, we built a variety of sequences needed to interact with the objects.

To give a practical example, think about moving a chair to sit in it, or to move it out of the way, or to grab a bottle of water and carry it without damaging the bottle itself. The ultimate goal of the system is being able to find or pick up the bottle if it was dropped, or assist in delivering the water bottle at the right moment and time, based on the user’s condition.

The amount of use cases is ever growing, so the only way to cover most of them is to deploy a system to run for a continuous period of time and use data to optimize for such sequences and experiences.

Another important thing to mention is to pay attention to the temperature mode of the devices one is using. If one is to run continuous ML models on the phones and Spot itself, they will find that the quality of processing can deteriorate dramatically because each respective operating system (OS) will try to throttle CPU/GPU cycles in an effort to reduce the temperature.

In perfect conditions, Spot’s battery should last for about 90 min. In our practical experiments, we were able to get 60 min of productive performance in the cooled down lab environment with the fully charged batteries. We were able to get about 30–40 min of productive performance in the room without AC running.

Quality of imagery sensors impacted the baseline of the computer vision models we trained using off-the shelf LIDAR and RGBD cameras provided by Boston Dynamics, as well as cameras (RGB, IR, LIDAR) provided by Apple iPhones and iPads, and Raspberry PI.

While building a high-definition mapping of the environment was critical for us to achieve a paper’s goal, we ultimately found building the proper number of loops inside the space became a bigger challenge, especially when the robot has to do that autonomously in the unknown environment for the first time (e.g., we changed the rooms), sometimes with the user who has less ability to provide context at hand, and ultimately leading to the robot making their first mistakes and self-correct with RLHF loop.

#### 3.10.2. Manipulation Model

We built a model inspired by the RT-1 model built on top of the transformers. However, we had to adjust the model to account for the mobility of the arm’s base—Ddog itself.

### 3.11. Communications

We designed the system with the assumption that at any point the system can be offline, and any of the parts can malfunction or error. Spot’s API allowed us to build fairly easy safety measures whenever Spot cannot communicate with any external systems, or when it comes to a needed outcome on its own.

Building safe autonomy is the goal of any project involving robotics, but it is also a challenge. We highly recommend making sure all components of the system can stop at any time and then pick up from any existing state.

#### 3.11.1. Wi-Fi vs. LTE

The majority of the data are exchanged between Ddog, Phone D, and Phone B using Wi-Fi that allows enough bandwidth for several streams in 1080p.

LTE is used to stream optimized 1080p using a high-efficiency video coding (HEVC) codec to reduce the amount of traffic at the expense of the higher energy consumption by Phone D.

Phone D’s LTE access is the main channel to communicate with Cloud D’s VPC, and Phone B’s LTE access is used to communicate with Cloud B’s VPCs.

#### 3.11.2. BLE

Paying attention to the energy consumption is critical across the system. BLE causes the least of the energy load on Phone B itself, and it is used only when the system tries to understand the user’s intent.

### 3.12. Energy Consumption

In perfect conditions, Spot’s battery can run for up to 90 min. With the charging system, the runtime can be extended to 4 h, which is a limit of the current battery system used in the AttentivU device. The phones can run for longer—in our case, the iPhones 12 and 13 Pro Max (Apple, Cupertino, CA, USA) were able to run ML and DL models for around 8 h before significant deterioration in the quality and temperature limits.

#### 3.12.1. Offline Mode

Each component of the system works without other parts of the system, providing more safety to implement various fail safes for multitude of reasons: environmental, hardware, network, user-related, etc.

Each component checks for the presence of the other components present on the network, so in case any of the components re-appear again (after reboot, battery re-placement, or loss of connectivity), the main components will try to reconnect and establish a new session with each component and device.

The autonomous controller is deployed on top of Phone D, as it can act as a single point of failure for the whole system. This risk should be avoided if the system will be deployed for continuous use by the users. The simplest solution would be to separate the layer of concern for the network (Wi-Fi hotspot) and the application itself, as well as to provide redundancy to the number of phones deployed, as it is fairly easy to achieve with the phone’s form factor.

#### 3.12.2. Telemetry

Because of the existing limitations with the connectivity, bandwidth, and energy consumption, we do not always relay the telemetry to Cloud B or Cloud D right away. All sensitive data are encrypted end-to-end (E2E) with the chain keys available to the Cloud VPCs. For production deployments, we will need to design reduced telemetry to collect aggregated data for the edge use-cases and also to support debugging of the system failures.

## 4. BCI Implementation and Results

### 4.1. AttentivU

**Figure 7:**
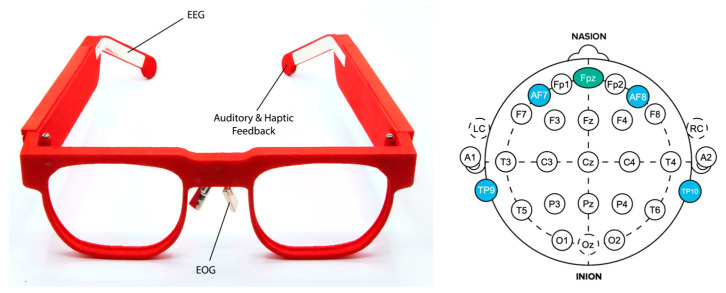
AttentivU glasses (**left**) and montage of EEG electrode locations (**right**). AttentivU glasses consisting of 2 EEG channels, TP9, and TP10, as well as a reference electrode at Fpz. It additionally has EOG channels and built-in audio and haptic feedback.

EEG data were collected from an AttentivU device. The AttentivU glasses feature electrodes made from natural silver located at the TP9 and TP10 locations according to the international 10–20 system of electrode placement. The glasses also consist of two EOG electrodes located at the nosepads and one EEG reference electrode located at the Fpz location (Figure 7). We refer the reader to [10,11] to learn more about the glasses’ architecture.

The main advantage is the portability of the device as well as the ease of use: the time spent to equip a user with the glasses is less than 2 min. The device also features two modalities (EEG and EOG), which are both extremely useful to capture a phenomenon at hand, e.g., an active mental task. EEG and EOG sensors are reported in multiple research papers as the signals that can be used to capture attention, engagement, fatigue, and cognitive load in real time. EEG has been used as a neurophysiological indicator of the transition between wakefulness and sleep [44,45,46] and has been tested in different environments where attention and vigilance are required [47,48]. EOG is based on measuring the biopotential signals that are induced due to cornea–retina dipole characteristics during eye movement. Researchers have shown that eye movements correlate with the type of memory access required to perform certain tasks and are good measures of visual engagement, attention, and drowsiness [49,50]. Various studies have indicated that signals from different modalities can represent different aspects of covert mental states [51,52,53,54]. EEG and EOG can represent internal cognitive states and external subconscious behaviors, respectively. These two modalities contain complementary information and when combined can be used to construct more robust estimation models of vigilance, attention, and engagement based on state-of-the-art methods [10]. In the context of our task, these sensors can provide the required information, as well as support real-time, closed-loop interventions when needed.

Additionally, several papers report using lateral posterior electrodes (our glasses’ EEG electrode location) and entropy measures for vigilance assessment. For example, Hu [55] used entropy measures for feature extraction from a single electroencephalogram (EEG) channel, reporting performance of around 90% of a single channel, achieved using a combination of a single channel among T6, TP7, T5, TP8, T4, and T3 with the combination of feature fuzzy entropy (FE) and random forest classifier (RF). Mu et al. [56] also used entropy measures for EEG signals collected in resting and fatigue states in order to extract features. Retrospective analysis of the EEG showed that the extracted features from electrodes T5, TP7, TP8, and FP1 yielded better performance.

T8 channel is a well-established region for detecting changes in the drowsiness/fatigue state [45,53,57]. Thus, we believe that electrodes TP9 and TP10 that are positioned in close proximity to T8 and other lateral posterior electrodes are well positioned to detect and replicate the aforementioned findings as well as focused states.

EOG signals provide information on various eye movements, which are often used to estimate vigilance because of an easy setup and high signal-to-noise ratio (SNR) [58,59]. In comparison with EEG, the amplitude of EOG is significantly higher, which makes EOG more robust to noise than EEG. Kanoh et al. [60] proposed placing only three electrodes for EOG measurement, which is similar to our setup. In their approach, three electrodes are used to measure EOG. One electrode is located on the nose bridge, and the other two electrodes are placed on left and right side nose pads, respectively.

Having two physiological modalities naturally leads to a question of feasibility of fusing both of them to gain into improved classification outcomes. In Khushaba et al. [45], for example, a feature-extraction algorithm was developed to extract the most relevant features to predict driver drowsiness and fatigue states by analyzing the corresponding physiological signals from the three EEG and one EOG channel as well as electrocardiogram (ECG). Using EEG channels alone or a combination of EEG + ECG or EEG + EOG were shown to achieve highly accurate classification results when using nonlinear feature-projection methods. Huo et al. [61] have shown that fusing forehead EOG data from 4 electrodes and EEG data from 12 electrodes can improve the performance of fatigue detection. The authors also report that modality fusion of EEG and EOG yielded models of higher prediction correlation coefficient and lower root mean square error (RMSE) value in comparison with signal modality (EEG or EOG).

We refer the reader to Kosmyna et al. [10], which contains these and more references, covering in more depth an overview of using EEG and EOG in the glasses form-factor.

We used our own custom pipeline to stream data from the glasses.

### 4.2. Pilot Study

**Figure 8:**
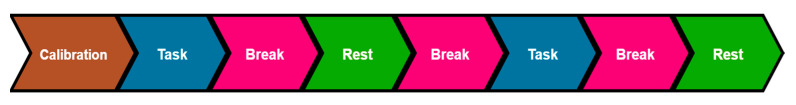
Task structure for a run. Here, task and rest were of a duration of 2 min each, whereas calibration and break were 40 s each.

Three subjects (2M/1F) were selected for this pilot study. One subject had ALS. The experiment paradigm (e.g., ‘run’) was an active, eye-free activity, where subjects either had to perform a mental task (see below) or mental relaxation (resting). Each of these were cascaded together and were separated by breaks, which were considered as buffer neutral states (Figure 8). The mental task and mental relaxation were of 2 min each, and every break was of a 40 s duration. We define a ‘run’ as a sequence of the following tasks in order: task, break, rest, break, task, break, rest. Every run started with a 40 s initialization period where the subjects had to minimize movements and try to be in a neutral state of mind.

#### 4.2.1. Tasks

We chose three tasks for the pilot study, namely, mental mathematics (MA), word association (WA), and melody (ME) [26]. In the mental mathematics task, the subject had to rapidly perform a series of mental calculations (moderate to high difficulty—depending on the comfort level of the subject) on random numbers, for example, (128 × 56), (5689 + 7854), (36 × 12). In the word association task, the subjects had to come up with words. They had to start with a random word, and then subsequently generate a word that started with the letter that was the previous word’s last letter, for example, elephant, tiger, or racoon. In the melody task, they had to think of a familiar tune and further imagine listening to it without articulating words but rather focusing on the melody itself.

#### 4.2.2. Methodology and Classification

**Figure 9:**
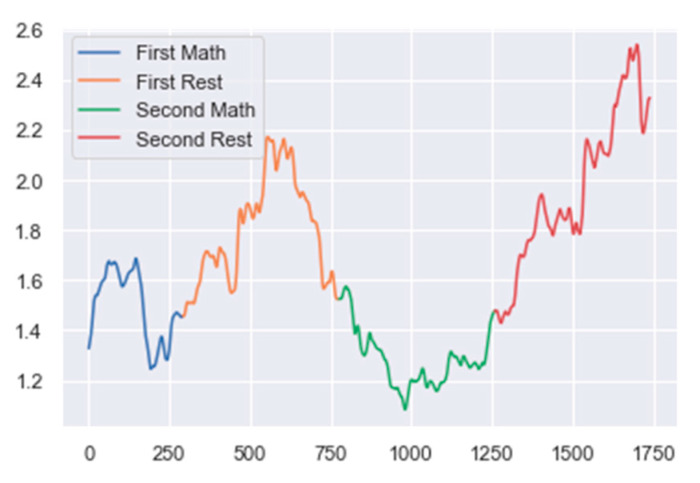
The above curve is the alpha/delta ratio, which is used to classify the MA task. The changes in this curve are used to determine when the subject changed their mental state.

First, the EEG data were split into epochs/windows. We define each window as a 1 s long duration of EEG data with a 75% overlap with its previous one. The next step was data preprocessing and cleaning. The epoched data were filtered using a combination of a 50 Hz notch filter and a bandpass filter with a passband of 0.5 Hz to 40 Hz that ensured power line noise and unwanted high frequencies were removed. Next, we created our own artifact rejection algorithm. The artifact rejection algorithm works by rejecting an epoch if the absolute power difference between two consecutive epochs is greater than a predefined threshold. In the final step of classification, we used a mix of different spectral band power ratios to track the mental activity of each subject based on the task. For MA, this ratio was (alpha/delta). For WA, this ratio was (delta/low beta), and for ME, this ratio was (delta/alpha) [26]. We then used a change point detection algorithm to keep track of changes to these ratios. A sudden increase or decrease in the magnitude of these ratios indicated a change in the mental state of the user. Whenever a possible change was detected, we checked if the change indicated a new class using an algorithm based on the difference in means of the ratios at the current and previous change. A change was considered valid if it led to a new class or changes the trend of the ratio. All other changes were considered invalid and rejected. Figure 9 represents this ratio for a run of the MA task. We also explored other supervised approaches that resulted in high testing and training accuracies, but the models performed poorly when used with real-time data. The proposed unsupervised approach never ‘trains’ on any data and constantly adapts to changes and shifts in band powers; hence, is expected to perform well in practical conditions.

#### 4.2.3. Results

The proposed model achieved accuracies of 73% for the MA task, 74% for WA task, and 60% for ME task for the subject with ALS. The same model achieved 72% for the MA task, 60% for the WA task, and 58% for the ME task on other subjects. It is worth noting that the other two subjects were able-bodied and had more body and eye movements compared to the subject with ALS. This could be a factor that can influence the quality of data and the obtained results. Table 1 contains results for each subject using an unsupervised approach that works best on real-time data and has a minimum classification time. Table 2 contains results using one of the many supervised approaches we explored; here, the results were generated using an LSTM model [54]. We thus decided to use MA as our task for the main study, but there seemed to be a lot of promise in using other mental brain-teaser tasks, like WA. 

### 4.3. Main Study

Two healthy subjects (1M/1F, median age 32 years old) participated in the study. Both subjects had prior experience with BCI. As mentioned earlier, the BCI paradigm was set as an active mental activity where the subjects either had to do a mental task or rest (REST). We chose MA as our active mental task.

**Figure 10:**
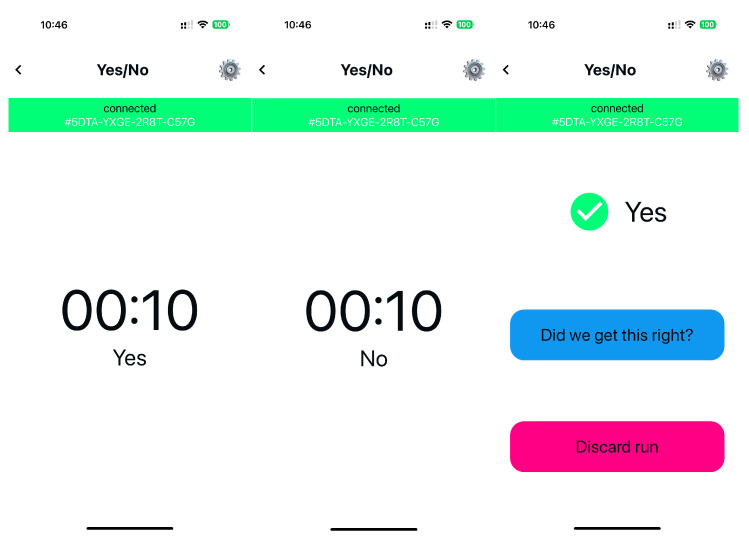
BioSignal application’s UI for a YES/NO choice. From left to right: countdown to select YES as a response; countdown to select NO as a response. Response YES is an output of the system.

As touched upon in the infrastructure section of this paper, we used a carefully architected data pipeline for handling EEG data, which formed the backbone of this research contribution. The pipeline comprises two essential segments: the frontend and the backend. The frontend includes an EEG device (AttentivU) and a client app installed on an iPhone (Figure 10). AttentivU records electrical activity of the brain and relays these data to the client app. This app is designed to offer a user-friendly interface, enabling users to start data collection recordings, observe recorded data, and initiate an inference mode to allow them to ask and respond to questions using a YES/NO option (described in more details below). The backend of the pipeline encompasses cloud-based components that facilitate effective data streaming, long-term storage, and real-time prediction. Google Cloud Platform Cloud Storage was utilized, enabling secure and cost-effective retention of substantial volumes of data. Machine learning (ML) models residing in the backend are another key component. They perform real-time predictions on the cached data, enabling various options for communication for the user. To guarantee the scalability and reliability of the BCI system, all backend components, barring cloud storage, were hosted in a Kubernetes cluster. This comprehensive infrastructure allows for gathering a significant volume of EEG signals from multiple users.

### 4.4. Task and BCI Sessions

Each subject performed a total of 30 runs of supervised sessions, which we call ‘robotic sessions’, where they were asked to provide a pre-defined mental response to one of six questions with a binary choice YES/NO, like ‘Would you like me to go to a kitchen?’ A text-to-speech voice as well as text on-screen were used to indicate the time periods and transition periods (see Figure 10). Each participant was asked to pick one of the options (YES or NO), and when they heard their choice, to perform a MATH task for 10 s; if it was not their choice, they were informed to perform REST for 10 s. An example of the run is as follows: a subject heard ‘Please respond with a YES to the following question: ‘Would you like me to go to a kitchen?’’. Once this instruction was played, the countdown to YES was played 3 s later for 10 s, during which the subject was supposed to perform mental calculations. There was then a 3 s break, and the word ‘NO’ was then played for 10 s; in this case, the subject was supposed to be in a resting state. If the classification output was YES, then Spot would move to the kitchen area, regardless of its current position. The robotic session included all the responses to be executed by Spot.

Though potentially tiring, this supervised nature of the robotic run was performed in order to evaluate the BCI output. Each question was repeated five times (with a total of 30 runs), and the questions asked included: ‘Would you like me to go to a kitchen?’, ‘Would you like me to go to a window?’, ‘Would you like me to go to a living room?’, ‘Would you like me to take a picture?’, ‘Would you like me to bring a toy?’, and Would you like me to bring a chair?’ There was a 5 min break after the 15th run. It is important to note that prior the robotic session, we demonstrated to the user ‘the intended response’ action from the robot when YES was selected for each of the questions, e.g., where and how the robot moves in response to the kitchen, living room, and window. Finally, the actions of taking a picture, bringing a toy, and bringing a chair were also demonstrated so the user would not be surprised or scared regarding what actions and sounds to expect from Spot in addition to moving around the room. When NO was selected, no movement or action was performed by Spot. We called this session a pet session. No activities were performed by the subject during pet session, they were just observing the robot.

A short training session of 10 runs preceded the actual ‘robotic’ session. Each run in a training session also included 10 s of recording for each option (MATH/REST), and a text-to-speech voice as well as text on-screen was used to provide users with feedback, e.g., screen and voice indicating YES or NO (YES/NO were requested beforehand by a system). There was a 5 min break between the training session and the robotic session. Spot was not used during the training session.

Finally, an unsupervised session concluded the task. It took place after a 5 min break once the robotic session was over. The unsupervised session included two runs for each question, but the subject decided how to respond to each question themselves. Spot was used here, similar to the robotic session, in order to execute the user’s responses. We asked the subject to inform us of their intended response after each run in order to label the responses. We finally conducted informal interviews with the subjects in order to collect their opinions about Ddog, and more specifically, regarding the ease of use, comfort, and effectiveness.

### 4.5. Results

The results can be seen in Table 3. As we only had two subjects, these results need to be considered as preliminary. Regarding the subjective opinions, both subjects reported the ease of use, comfort, and overall satisfaction of the system. They mentioned that they enjoyed controlling the robot and that they were highly motivated to succeed in their control, especially during the unsupervised session.

## 5. Challenges, Limitations, and Future Work

The majority of the challenges in the space of robotics and human interaction come from the safety hazards and technological constraints, as well as the constraints of the AI agency and its autonomy in making its own decisions and interacting with the environment and humans. Technical challenges that will be addressed over time are dependent on the energy consumption as well as battery form factor and cost. The quality of the imagery sensors will increase over time to collect information about the environment in real time at a higher degree of control and quality, so the robotics system can be made more aware of the context and the environment.

As already mentioned, the Spot robot was designed for industrial use cases, and applying the same hardware to the assistant use cases and home/office environment is feasible but potentially not ideal. Thus, developing guides for at-home navigation and interaction requires further research and experimentation, especially when the robots engage with humans directly.

Overall, our study should be considered as a proof of concept (PoC) and all the findings treated as preliminary as we only had two healthy subjects; thus, any statistical analysis would not be possible. The subjects participated in our studies pointed out that they felt a high engagement from both controlling a robot and also using a BCI system to do so. We believe that more studies involving both healthy controls but also patients with LIS/ALS should be performed. The BCI paradigm used should also be further evaluated, as though no decline in performance or decline resulting from habituation seemed to have occurred as highlighted by other research works using mental calculations [26], personalized models should be explored further, as well as other ‘brain-teaser’ paradigms like word association.

As pointed out by Spataro et al. [29], besides the potential economic benefits of reducing assistive needs, the control of a robotic assistant could be of a significant psychological significance, restoring basic forms of independence. Beyond helping with daily life tasks, the possibility of fostering positive emotions could offer a range of potential applications in rehabilitation and care for emerging robots [29].

Since Ddog can be programmed to perform an extremely broad spectrum of functions, our early results possibly pave the way towards new applications of brain-powered robotics to improve the autonomy of users. Since the applications of exploring robots in human tasks are rapidly expanding while costs are declining, this study might pioneer the development of advanced robotic assistants.

## Figures and Tables

**Table 1:** Results of the proposed model on MA, WA, and ME tasks.

Subjects/Task	Mental Mathematics (MA)	Word Association (WA)	Melody (ME)
Subject 1	73%	74%	60%
Subject 2	72%	60%	59%
Subject 3	64%	58%	56%

**Table 2:** Results obtained using the LSTM model.

Subjects/Task	Mental Mathematics (MA)			Word Association (WA)			Melody (ME)		
Subject 1	Training	Testing	Real Time	Training	Testing	Real Time	Training	Testing	Real Time
	90%	88%	65%	94%	92%	66%	86%	84%	62%
Subject 2	92%	91%	62%	94%	93%	62%	93%	92%	59%
Subject 3	91%	89%	66%	83%	82%	64%	87%	85%	59%

**Table 3:** Preliminary results. Data are expressed as ^1^ median with interquartile ranges; ^2^ mean ± standard deviation.

Session Name	Training	Robotic	Unsupervised
Correct commands ^1^	10 (10–10)	30 (30–30)	12 (12–12)
% success ^2^	100 ± 0.0	100 ± 0.0	100 ± 0.0
% accuracy ^2^	74.5 ± 6.9	100 ± 0.0	83.4 ± 9.2

## Data Availability

Please contact the corresponding author.
